# Sodium (±)‐5‐bromo‐2‐(α‐hydroxypentyl) benzoate ameliorates pressure overload‐induced cardiac hypertrophy and dysfunction through inhibiting autophagy

**DOI:** 10.1111/jcmm.14468

**Published:** 2019-06-20

**Authors:** Bo Wang, Deliang Shen, Junnan Tang, Jing Li, Yue Xiao, Xiuying Chen, Chang Cao, Dongjian Han, Erhe Gao, Wen Zhao, Jinying Zhang, Junbiao Chang

**Affiliations:** ^1^ Department of Cardiology, Henan Province Key Laboratory of Cardiac Injury and Repair The First Affiliated Hospital of Zhengzhou University Zhengzhou P. R. China; ^2^ Key Laboratory of Advanced Pharmaceutical Technology, Ministry of Education of China, School of Pharmaceutical Sciences Zhengzhou University Zhengzhou P. R. China; ^3^ Center for Translational Medicine Temple University School of Medicine Philadelphia Pennsylvania; ^4^ School of Chemistry and Molecular Engineering Zhengzhou University Zhengzhou P. R. China

**Keywords:** autophagy, brozopine, cardiac hypertrophy, transverse aortic constriction

## Abstract

Sodium (±)‐5‐bromo‐2‐(a‐hydroxypentyl) benzoate (generic name: brozopine, BZP) has been reported to protect against stroke‐induced brain injury and was approved for Phase II clinical trials for treatment of stroke‐related brain damage by the China Food and Drug Administration (CFDA). However, the role of BZP in cardiac diseases, especially in pressure overload‐induced cardiac hypertrophy and heart failure, remains to be investigated. In the present study, angiotensin II stimulation and transverse aortic constriction were employed to induce cardiomyocyte hypertrophy in vitro and in vivo, respectively, prior to the assessment of myocardial cell autophagy. We observed that BZP administration ameliorated cardiomyocyte hypertrophy and excessive autophagic activity. Further results indicated that AMP‐activated protein kinase (AMPK)‐mediated activation of the mammalian target of rapamycin (mTOR) pathway likely played a role in regulation of autophagy by BZP after Ang II stimulation. The activation of AMPK with metformin reversed the BZP‐induced suppression of autophagy. Finally, for the first time, we demonstrated that BZP could protect the heart from pressure overload‐induced hypertrophy and dysfunction, and this effect is associated with its inhibition of maladaptive cardiomyocyte autophagy through the AMPK‐mTOR signalling pathway. These findings indicated that BZP may serve as a promising compound for treatment of pressure overload‐induced cardiac remodelling and heart failure.

## 1 INTRODUCTION

Cardiac hypertrophy is usually considered to be a compensatory response to mechanical and neurohumoral stimuli. In the early stages, hypertrophy of cardiomyocytes and thickening of the myocardium contribute to improvements in contractile function. However, the continued presence of pathological stress often leads to myocardial interstitial fibrosis, contractile depression and ventricular dilatation, ultimately resulting in heart failure 1, 2 and sudden death.^3^, ^4^ Therefore, cardiac hypertrophy is currently considered to be an independent risk factor for cardiovascular events.

Autophagy is a highly conserved lysosomal degradation pathway that helps to maintain cellular homoeostasis.^5^ Recently, accumulating evidence 6, 7 has suggested that autophagy plays an important role in the pathogenesis of cardiac remodelling and heart failure. Although basal autophagy is adaptive and beneficial, excessive or persistent autophagy can be maladaptive and harmful.^8^ Numerous studies 6, 7, 9 suggest that autophagy may be triggered in response to pathological stresses, such as pressure overload, and excess of such stresses may lead to cell death.^10^ Thus, regulating autophagy could be important for treating pathological cardiac remodelling and heart failure.

Derived from the natural compound apigenin, sodium (±)‐5‐bromo‐2‐(a‐hydroxypentyl) benzoate (generic name: brozopine, BZP) is a potential cerebrovascular and cardiovascular drug that was approved for clinical trials for stroke treatment by the China Food and Drug Administration (CFDA).^11^ BZP exhibits potent neuroprotective effects against ischaemic stroke, which may be related to its antioxidative effects 12 and inhibition of apoptosis.^13^ As is well‐known, autophagy can be induced by oxidative stress,^14^, ^15^ and antioxidants play a protective role in pressure overload‐induced cardiac hypertrophy.^16^, ^17^, ^18^ Besides, an increasing number of studies have revealed that crosstalk exists between autophagic and apoptotic machinery.^19^, ^20^ Therefore, it is reasonable to hypothesize that BZP may affect pressure overload‐induced hypertrophy and autophagy.

In the present study, we investigated the effect of BZP on cardiac remodelling and autophagy regulation with regard to the hypertrophic response. We found that BZP could inhibit angiotensin II (Ang II)‐induced cardiomyocyte hypertrophy and excessive autophagy through AMP‐activated protein kinase (AMPK)‐mammalian target of rapamycin (mTOR) signalling and could ameliorate pressure overload‐induced cardiac hypertrophy and heart dysfunction.

## 2 MATERIALS AND METHODS

### 2.1 Experimental animals and transverse aortic constriction (TAC) surgery

Animal protocols were approved by the Ethics Committee of Zhengzhou University. Male C57/BL6 mice (8 weeks old, 17‐22 g) were purchased from Beijing Vital River Laboratory Animal Technology Co. Ltd. (Beijing, China). The animals were housed at room temperature under a 12 hours light/dark cycle and provided a normal diet and purified water ad libitum. Animals were acclimatized to the laboratory environment for at least 7 days before the experiments.

Mice were subjected to TAC or a sham operation under anaesthesia (isoflurane, inhalation) as previously described.^21^ After anaesthesia and artificial ventilation were initiated, the transverse aorta was constricted by ligating the aorta with a 7‐0 nylon suture around a blunted 27‐gauge needle. The needle was removed immediately after the procedure. Mice in the sham group underwent all operation procedures except for the ligation. The effectiveness of aortic constriction was confirmed by performing echocardiography, and only mice with a pressure gradient over the aortic ligature ranging from 50 to 70 mm Hg were used in further experiments.

BZP bulk drug (purity 99.4%) was synthesized at the College of Chemistry and Molecular Engineering, Zhengzhou University (Zhengzhou, China). After the establishment of the TAC model, animals were treated with 20 mg/kg BZP or vehicle control by oral gavage once a day for 10 weeks.

### 2.2 Echocardiography analysis

To measure global cardiac function, echocardiography was performed at baseline and at 2, 4 and 10 weeks after TAC. Echocardiography was performed with an ultrasonic echocardiographic system (Vevo2100, VisualSonics Inc, Toronto, Canada). Briefly, after the chests of mice were shaved, the mice were fixed and underwent two‐dimensional echocardiography without anaesthesia. All parameters were evaluated by calculating the average of five cardiac cycles.

### 2.3 Cell culture and treatment

Neonatal rat cardiomyocytes (NRCMs) were isolated from 1‐ to 3‐day‐old Sprague‐Dawley (SD) rats as described previously.^22^ NRCMs were grown in DMEM supplemented with 10% FBS and 100 μM 5‐bromodeoxyuridine for 48 hours. Embryonic rat heart‐derived H9c2 cells were cultured in DMEM supplemented with 10% FBS. To induce hypertrophy, NRCMs or H9c2 cells were treated with Ang II (1 μM) for 48 hours. For other experiments, BZP (10 μM, 50 μM, or 250 μM), LY294002 (LY02) (5 μM) or Compound C (5 μM) was added at 2 hours before addition of Ang II, and the cells were incubated for 48 hours. Metformin (10 mM) was added for 0.5 hours before addition of BZP (250 μM). After treatment, the cells were harvested for analysis. LY294002, Compound C and metformin were purchased from MedChemExpress.

### 2.4 Histological analysis

Following anaesthesia, the heart was excised and immediately placed in 4% paraformaldehyde at room temperature for 24 hours. The myocardial specimens were embedded in paraffin and cut into 4 μm sections. Serial heart sections were stained with haematoxylin and eosin (H&E) or wheat germ agglutinin (WGA) to measure myocyte cross‐sectional areas (CSAs). The degree of cardiac fibrosis was detected by Masson's trichrome staining. The fibrotic areas were stained blue, and normal tissues were stained red. Images were analysed using a quantitative digital image analysis system (Image‐Pro Plus 6.0).

### 2.5 Electron microscopy

Cardiac tissue was cut into 1 mm cubes immediately after the heart was excised. Tissue blocks or H9c2 cells were fixed with 2.5% glutaraldehyde in 0.1 M phosphate buffer (pH 7.4) overnight at 4°C. After fixation, the selections were immersed in 1% buffered osmium tetroxide for 2 hours. The specimens were then dehydrated through a graded ethanol series and embedded in epoxy resin. After that, the specimens were sliced into ultrathin sections (60‐70 nm) with an ultramicrotome and post‐stained with uranyl acetate and lead citrate. Then, the sections were examined under an H‐800 electron microscope (Hitachi, Tokyo, Japan).

### 2.6 Immunofluorescence confocal microscopy

For immunofluorescence analysis of NRCMs, cultured NRCMs were fixed with 4% paraformaldehyde for 15 minutes, permeabilized with 0.1% Triton X‐100 in PBS for 10 minutes, blocked with 3% BSA solution for 1 hour and incubated with an anti‐actin (α‐sarcomeric) antibody (Sigma‐Aldrich; 1:200) or a microtubule‐associated protein 1 light chain 3 (LC3) A/B antibody (Cell Signaling Technology; 1:200) overnight at 4°C. Then, the cells were washed and stained with fluorescence‐conjugated secondary antibodies (Beyotime Biotechnology; Alexa Fluor 647 or Alexa Fluor 488, respectively). Immunofluorescence was analysed with a Nikon A1 confocal microscope (Nikon Corporation), and the cell surface areas and numbers of LC3 puncta were measured using Image‐Pro Plus 6.0 software.

### 2.7 Quantitative real‐time PCR (qRT‐PCR) and Western blot analysis

Total RNA in NRCMs and tissues was extracted using TRIzol reagent (Invitrogen), and first‐stand cDNA was synthesized using a RevertAid First Strand cDNA Synthesis Kit (Thermo Scientific). qRT‐PCR was performed with FastStart Universal SYBR Green Master (Roche) to examine the relative mRNA levels of the indicated genes as previously described.^23^ The sequences of the qRT‐PCR primers are shown in Table 1.

**Table 1:** The primer sequences for qRT‐PCR

Genes	Sequences
mouse ANF	F:TTCAAGAACCTGCTAGACCACC R:GCGAGCAGAGCCCTCAGTTT
mouse BNP	F:GCTGCTGGAGCTGATAAGAGAA R:CGATCCGGTCTATCTTGTGCC
mouse β‐MHC	F:ACGGATAGCGCCTTTGACG R:TACTCGTTGCCCACTTTGACTC
mouse GAPDH	F: CCTCGTCCCGTAGACAAAATG R:TGAGGTCAATGAAGGGGTCGT
rat ANF	F:CAAGAACCTGCTAGACCACC R:AGCCCTCAGTTTGCTTTTCA
rat BNP	F:CAGTCTCCAGAACAATCCACGA R:CTAAAACAACCTCAGCCCGTCA
rat GAPDH	F:CATCATCCCTGCATCCACTG R:GCCTGCTTCACCACCTTCTT

For Western blot analysis, heart tissues and NRCMs were lysed in RIPA buffer (Solarbio). Proteins were isolated as previously described,^24^ and the lysates (30 μg) were subjected to 10% SDS‐PAGE and transferred to NC membranes. The membranes were then incubated with specific antibodies against different antigens overnight at 4°C. Then, the membranes were incubated with a horseradish peroxidase‐conjugated secondary antibody (ZSGB‐BIO) and visualized with X‐ray film. The signals were quantified using ImageJ software. Antibodies against LC3 A/B, Beclin‐1, mTOR, p‐mTOR (Ser2448), AMPKα and p‐AMPKα (Thr172) were purchased from Cell Signaling Technology. An antibody against p62 was purchased from Proteintech, and an antibody against β‐actin was purchased from ZSGB‐BIO.

### 2.8 Statistical analysis

Data are expressed as mean ± standard error of the mean (SEM) from at least three independent experiments. Student's *t* test was performed for analysis of two groups and one‐way ANOVA followed by Newman‐Keuls multiple comparison test for three or more groups. *P* < 0.05 was considered statistically significant.

## 3 RESULTS

### 3.1 Ang II‐induced hypertrophy and autophagy in cardiomyocytes

**Figure 1:**
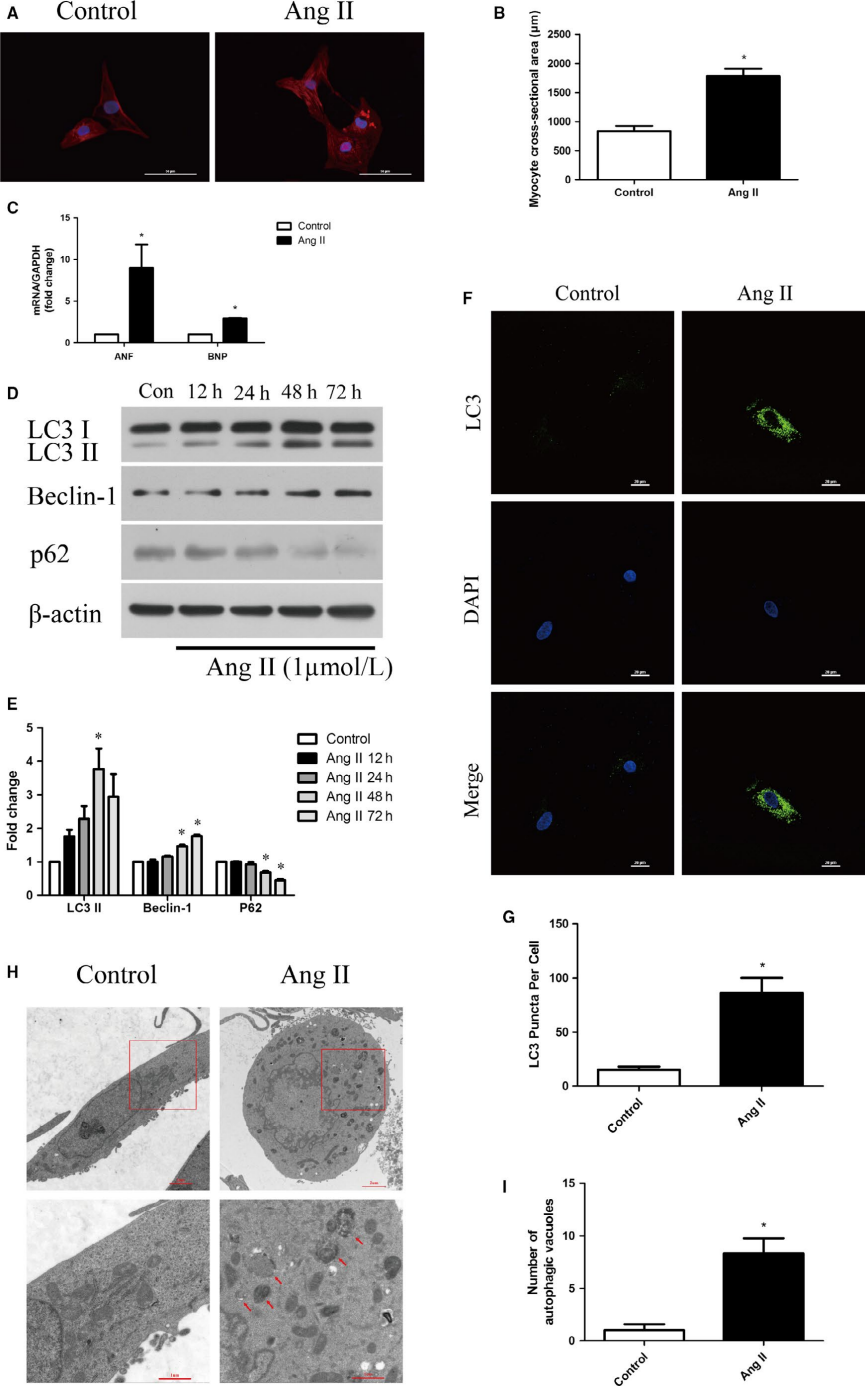
Ang II‐induced hypertrophy and autophagy in cardiomyocytes. (A‐B) NRCMs were treated with Ang II (1 μM) for 48 h. α‐Sarcomeric actin staining was performed to determine cell size. Representative images and the quantification of cell size (n = 20 cells) are shown. (C) NRCMs were treated as shown above, and qRT‐PCR was performed to analyse the mRNA levels of hypertrophic genes (ANF, BNP) (n = 3). (D‐E) NRCMs were treated with Ang II (1 μM) for 12, 24, 48 and 72 h. LC3 I/II, Beclin‐1, and p62 are shown in Western blots and are presented in a bar graph (n = 3). (F‐G) NRCMs were treated as shown in (A). Endogenous LC3 puncta were observed by immunofluorescence and the number of LC3 puncta per cell was quantified (n = 20 cells). (H‐I) H9c2 cells were treated as shown in (A). Representative transmission electron microscopy images of the autophagic ultrastructure are shown and the number of autophagic vacuoles per cell was quantified. The data are expressed as the mean ± SEM. *P ＜ 0.05, compared to the Control group

NRCMs were incubated with Ang II (1 μM) for 48 hours to induce cardiomyocyte hypertrophy. α‐Sarcomeric actin staining and qRT‐PCR were performed to validate the cardiomyocyte hypertrophic model. As shown in Figure 1A,1, Ang II stimulation significantly increased cell surface area. The average cell surface area in Ang II group was twice that in Control group. In addition, the qRT‐PCR data (Figure 1C) revealed that Ang II treatment clearly increased the mRNA expression levels of atrial natriuretic factor (ANF) and B‐type natriuretic peptide (BNP), which are hypertrophic genes.

As shown in Figure 1D,1, NRCMs were incubated with Ang II (1 μM) for 12, 24, 48 and 72 hours. Western blotting assay showed that Ang II stimulation increased the expression of Beclin‐1, and decreased the expression of p62 in a time‐dependent manner. LC3 II expression increased at 12 hours, peaked at 48 hours, and decreased at 72 hours after Ang II stimulation. In addition, a characteristic of autophagy is the recruitment of LC3 to autophagic vesicles. The immunofluorescence staining assay showed that endogenous LC3 puncta markedly increased after Ang II stimulation (Figure 1F,G). In agreement with the above results, microscopic images showed that Ang II stimulation increased the number of autophagic vacuoles (Figure 1H,I).

These data indicated that Ang II stimulation obviously induced cardiomyocyte hypertrophy and triggered autophagy.

### 3.2 BZP attenuated Ang II‐induced autophagy in cardiomyocytes

**Figure 2:**
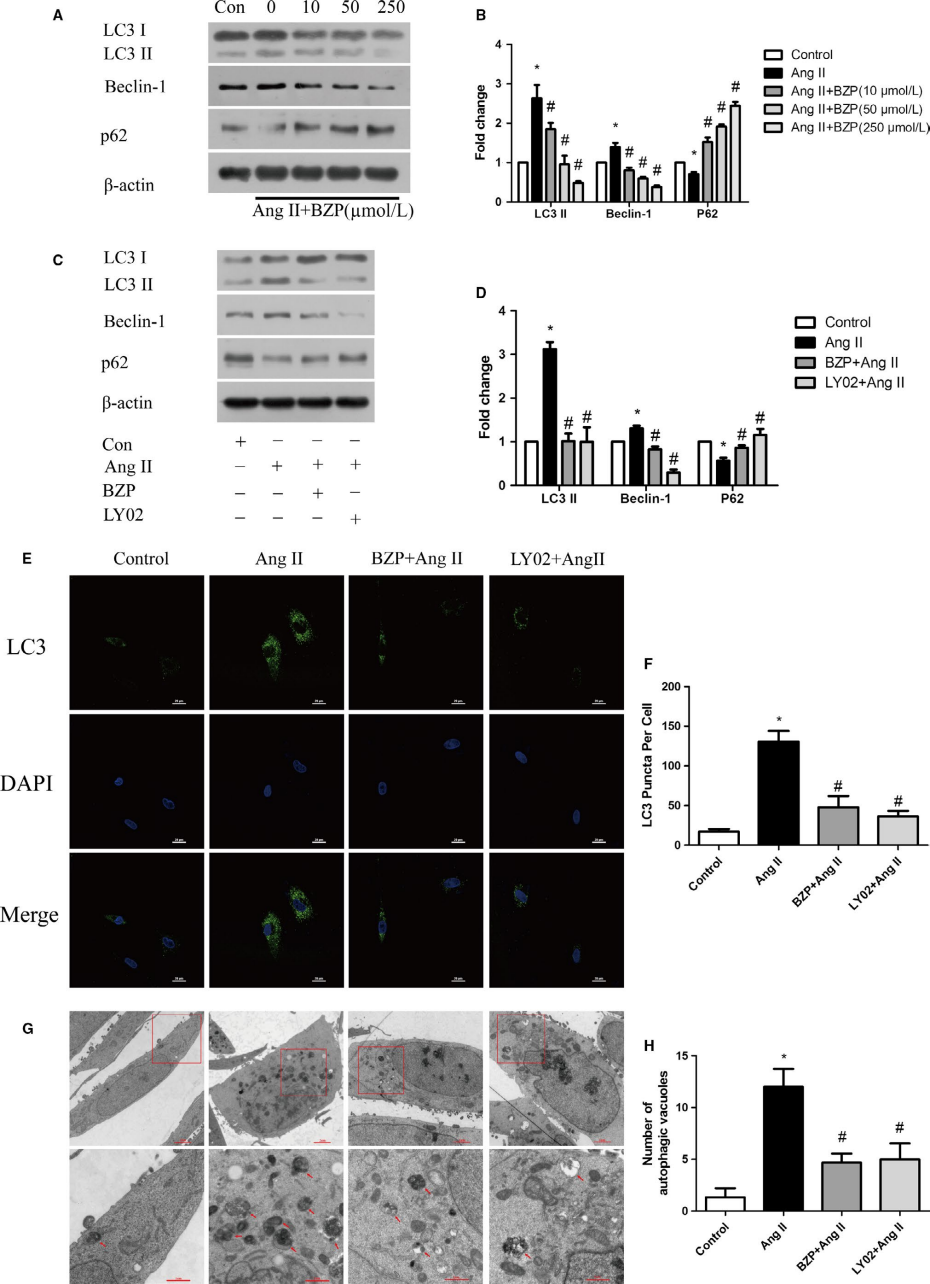
BZP attenuated Ang II‐induced autophagy in cardiomyocytes. (A‐B) NRCMs were pre‐treated with different concentrations of BZP (10 μM, 50 μM, 250 μM) for 2 h and exposed to Ang II (1 μM) for 48 h. LC3 I/II, Beclin‐1, and p62 are shown in Western blots and are presented in a bar graph (n = 3). (C‐D) NRCMs were pre‐treated with or without BZP (250 μM) or LY294002 (5 μM) for 2 h and exposed to Ang II (1 μM) for 48 h. LC3 I/II, Beclin‐1, and p62 are shown in Western blots and are presented in a bar graph (n = 3). (E‐F) NRCMs were treated as shown in (C). Endogenous LC3 puncta were observed by immunofluorescence and the number of LC3 puncta per cell was quantified (n = 20 cells). (G‐H) H9c2 cells were treated as shown in (C). Representative transmission electron microscopy images of the autophagic ultrastructure are shown and the number of autophagic vacuoles per cell was quantified. The data are expressed as the mean ± SEM. *P ＜ 0.05, compared to the Control group; ^#^ *P* ＜ 0.05, compared to the Ang II group

To investigate the effects of BZP on Ang II‐induced autophagy, we performed Western blotting, immunofluorescence staining and electron microscopy. As shown in Figure 2A,2, exposure to BZP decreased the elevated LC3 II and Beclin‐1 levels induced by Ang II in a dose‐dependent manner in NRCMs. Moreover, a marked increase in p62 was also observed. Next, we used LY294002 as a positive control and examined the effect of BZP on Ang II‐induced autophagy. As expected, there was significantly lower protein expression of LC3 II, Beclin‐1 and higher expression of p62 in the BZP treatment group and in the LY294002 treatment group than in the Ang II‐only group (Figure 2C,2).

Consistent with the results of the Western blotting assay, the immunofluorescence staining (Figure 2E,2) and microscopic images (Figure 2G,H) showed similar trends. These data indicated that Ang II‐induced autophagy was significantly suppressed by BZP.

### 3.3 BZP ameliorated Ang II‐induced hypertrophy in NRCMs

**Figure 3:**
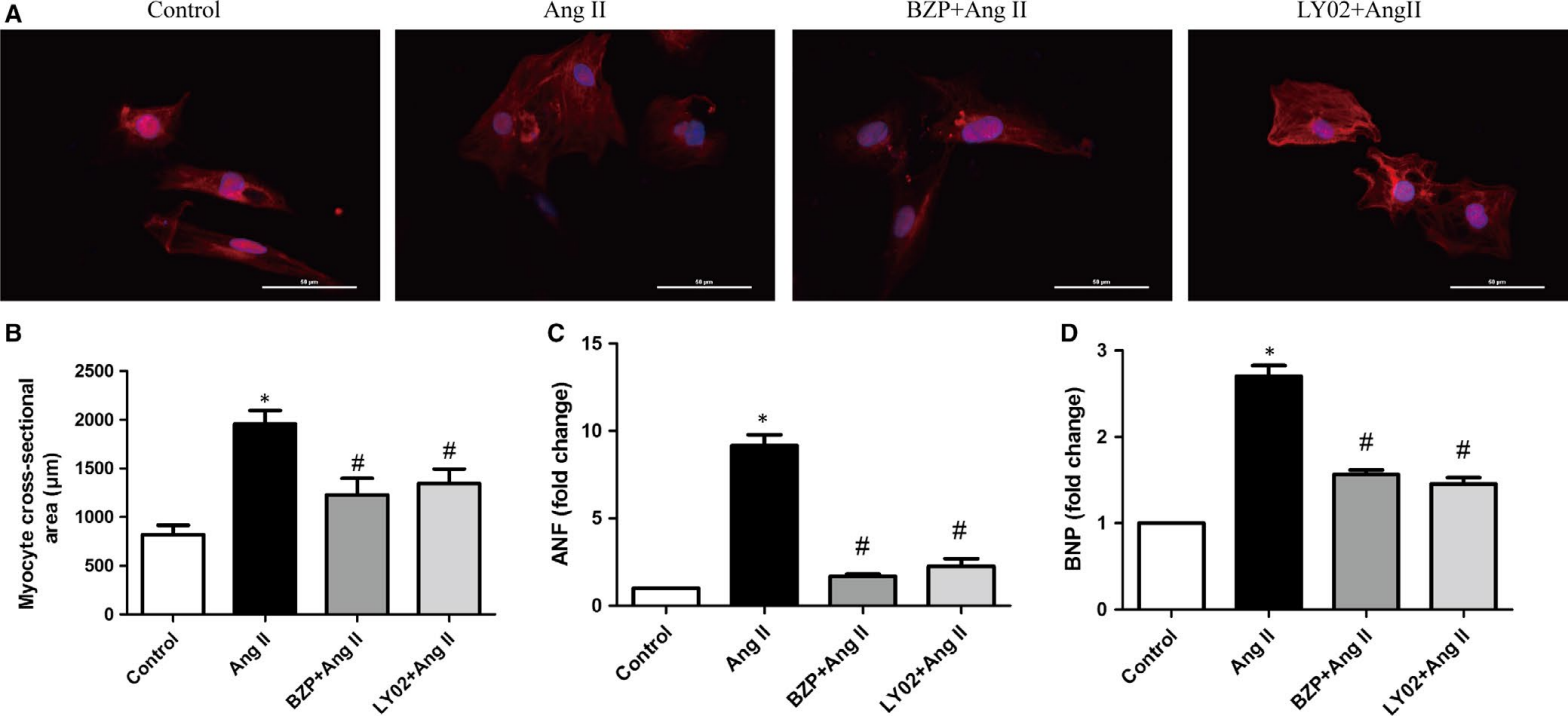
BZP ameliorated Ang II‐induced hypertrophy in NRCMs. (A‐B) NRCMs were pre‐treated in the presence or absence of BZP (250 μM) or LY294002 (5 μM) for 2 h and exposed to Ang II (1 μM) for 48 h. α‐Sarcomeric actin staining was performed to determine cell size. Representative images and the quantification of cell size (n = 20 cells) are shown. (C‐D) NRCMs were treated as shown above, and qRT‐PCR was performed to analyse the mRNA levels of hypertrophic genes (ANF, BNP) (n = 3). The data are expressed as the mean ± SEM. *P ＜ 0.05, compared to the Control group; ^#^ *P* ＜ 0.05, compared to the Ang II group

To determine whether BZP could ameliorate cardiomyocyte hypertrophy induced by Ang II, we performed immunofluorescence staining and qRT‐PCR. As shown in Figure 3A,3, cell surface area was dramatically increased by Ang II treatment, but BZP significantly prevented this increase. In addition, we used the autophagy inhibitor LY294002 as a positive control and found that LY294002 treatment also hindered the enlargement of cell size.

Next, the qRT‐PCR data revealed that Ang II treatment clearly increased the mRNA expression levels of ANF and BNP, while BZP or LY294002 treatment reduced the Ang II‐induced increases in the mRNA expression of these genes (Figure 3C,D).

### 3.4 BZP alleviated TAC‐induced cardiac hypertrophy in mice

**Figure 4:**
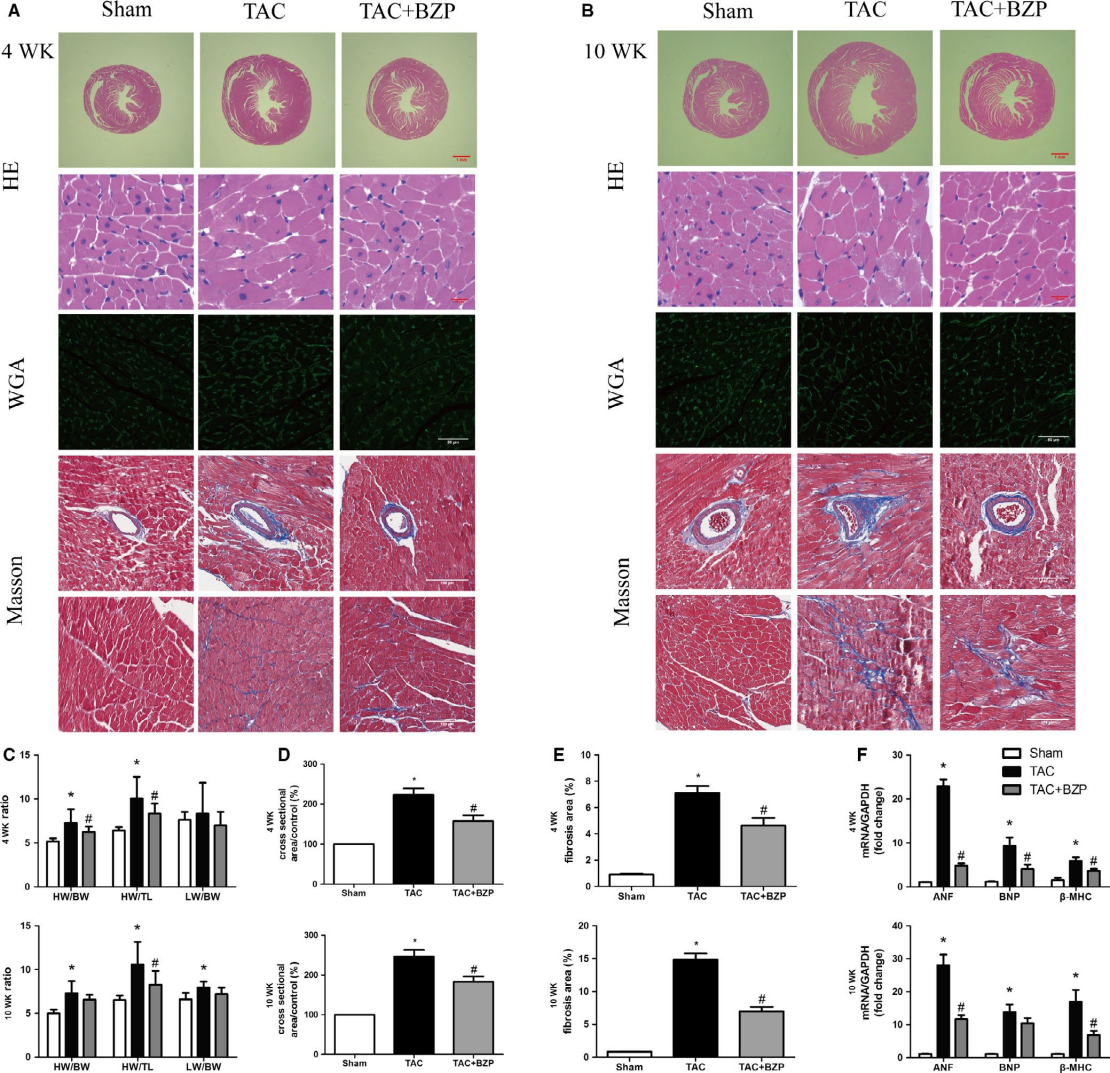
BZP alleviated TAC‐induced cardiac hypertrophy in mice. (A‐B) Representative images of heart cross sections and cardiomyocytes with H&E staining, WGA staining and Masson's staining at 4 wk (A) and 10 wk (B) after TAC surgery with or without BZP treatment. (C) Statistical results of HW/BW, HW/TL and LW/BW ratios (n = 12 at 4 wk and n = 5‐8 at 10 wk after TAC surgery). (D) Quantification of cardiomyocyte CSA (n = 100 cells per section). (E) Quantification of the percent of fibrotic tissue (n = ~20 images from 4 hearts per group, ~5 images per heart). (F) Quantification of qRT‐PCR data for hypertrophic markers including ANF, BNP, β‐MHC from heart tissues of different groups (n = 3,4). The data are expressed as the mean ± SEM. *P ＜ 0.05, compared to the Sham group; ^#^ *P *＜ 0.05, compared to the TAC group

To investigate the in vivo effects of BZP on cardiac hypertrophy, we established an animal model by performing TAC surgery. Four weeks of TAC caused significant hypertrophy in the C57BL/6 mice. At 4 weeks after TAC surgery, the heart weight/body weight (HW/BW) and heart weight/tibia length (HW/TL) ratios were significantly lower in the BZP‐treated group than in the TAC‐only group. However, the lung weight/body weight (LW/BW) ratios did not vary among the three groups (Figure 4C). In addition, the H&E staining and WGA staining results for myocyte CSAs also confirmed the protective effects of BZP against TAC‐induced hypertrophy (Figure 4A,D). Subsequent analysis of the mRNA expression levels of hypertrophic genes showed similar trends (Figure 4F).

Cardiac hypertrophy at 10 weeks after TAC surgery was more severe than that at 4 weeks after TAC surgery (Figure 4A,4). After 10 weeks of TAC, we again observed that BZP significantly decreased the HW/TL ratios of the BZP‐treated group compared to the TAC‐only group (Figure 4C). The therapeutic effects of BZP on TAC‐induced hypertrophy were further demonstrated by H&E staining, WGA staining and mRNA expression analysis of hypertrophic markers (Figure 4A,4,4).

Masson's staining indicated that pressure overload induced progressive interstitial fibrosis in the TAC‐only group. However, BZP administration significantly attenuated the extent of myocardial fibrosis at both 4 weeks and 10 weeks after TAC surgery (Figure 4A,4,4).

### 3.5 BZP ameliorated TAC‐induced cardiac dysfunction in mice

**Figure 5:**
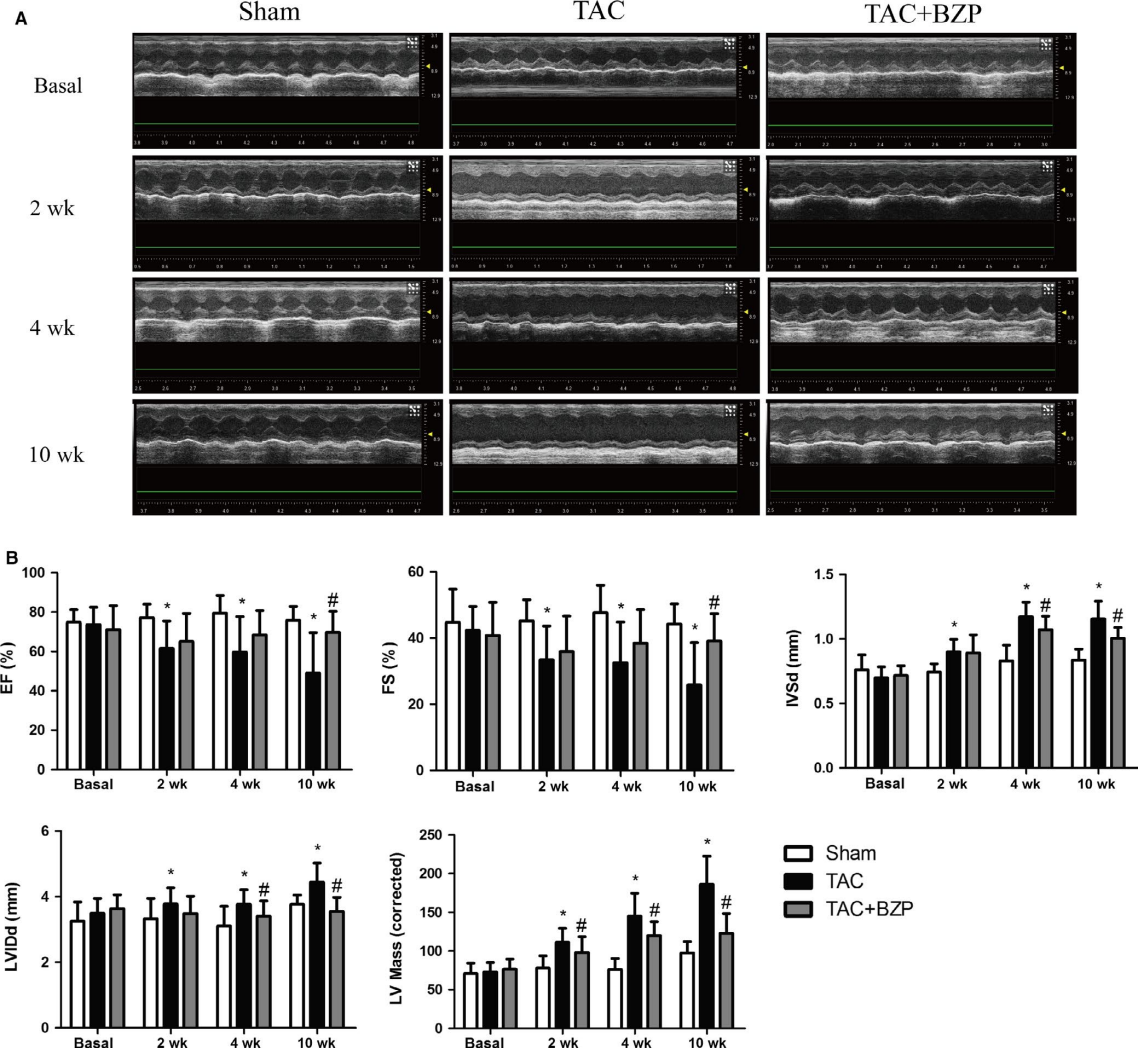
BZP ameliorated TAC‐induced cardiac dysfunction in mice. (A) Representative echocardiography of mice at baseline and 2, 4, and 10 wk after TAC surgery with or without BZP treatment. (B) Quantification of echocardiographic parameters (n = 17‐20 at baseline and 2 and 4 wk after TAC, n = 5‐8 at 10 wk after TAC). The data are expressed as the mean ± SEM. *P ＜ 0.05, compared to the Sham group; ^#^ *P *＜ 0.05, compared to the TAC group

To gain further insight into the effects of BZP, we evaluated cardiac function in the three groups of mice at different time points. A few representative short‐axis views from each group are shown (Figure 5A). According to echocardiographic evaluation (Figure 5B), the TAC‐only group started to show significant reductions in ejection fraction (EF) and fractional shortening (FS) compared to the sham group at 2 weeks after TAC surgery, and these effects became aggravated over time. At 4 weeks after surgery, compared to the sham group, the TAC‐only group showed significant decreases in EF (from 79.52% to 59.73%) and FS (from 47.68% to 32.59%). Treatment with BZP improved EF (to 68.54%) and FS (to 38.49%). At 10 weeks after surgery, compared to the values at 4 weeks, EF and FS in the TAC group had declined further (to 49.10% and 25.86%, respectively), but these two parameters remained unchanged in the BZP‐treated group. In addition, the geometrical parameters of the heart, including the interventricular septal thickness at end‐diastole (IVSd), left ventricular (LV) internal dimension at end‐diastole (LVIDd) and LV mass (corrected), were also significantly improved in the BZP‐treated mice (*P* < 0.05) compared to the TAC‐only mice.

### 3.6 BZP attenuated TAC‐induced excessive autophagic activity in the myocardium in mice

**Figure 6:**
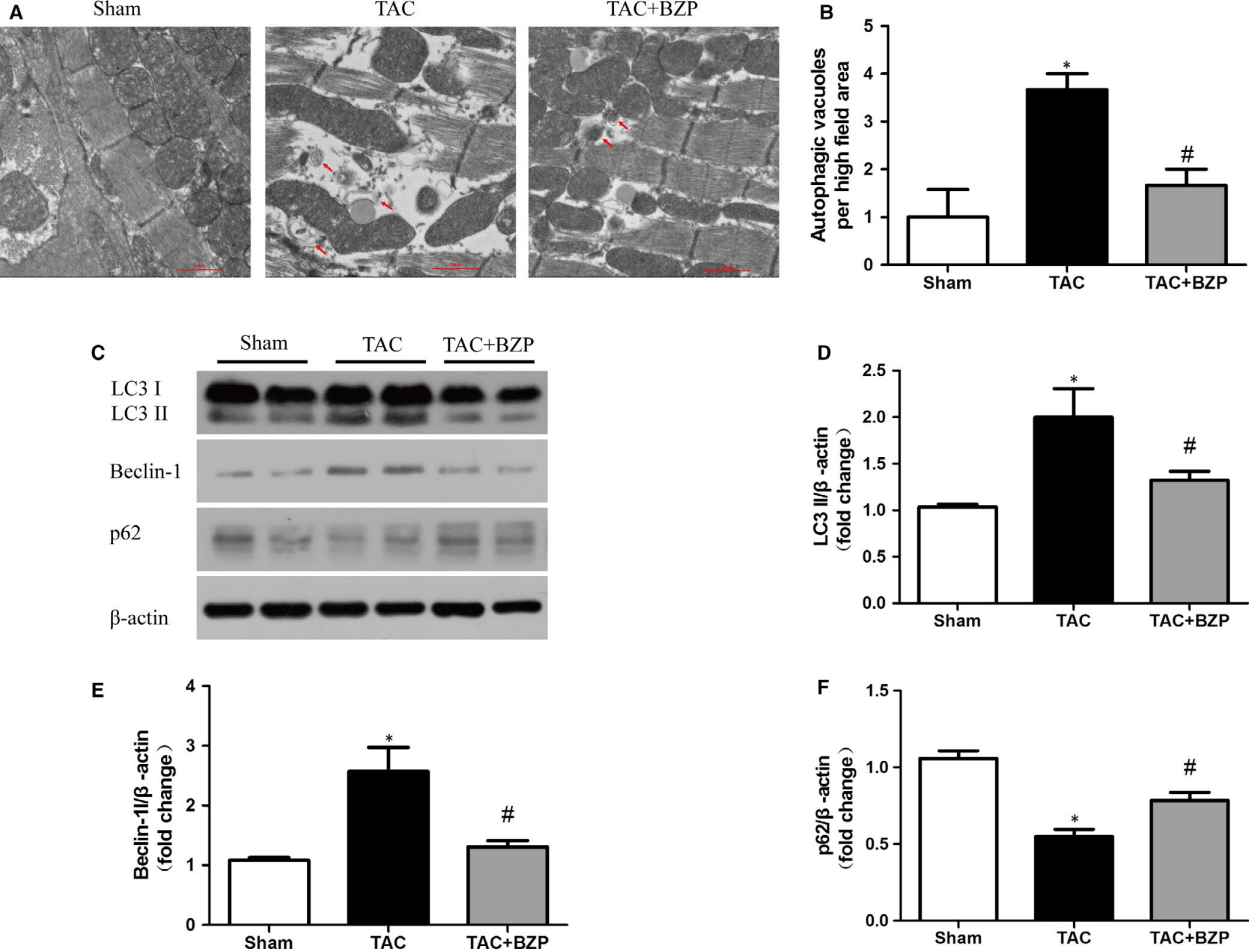
BZP attenuated TAC‐induced excessive autophagic activity in the myocardium in mice. (A) Representative transmission electron microscopy images of the autophagic ultrastructure of heart tissue at 10 wk after TAC. Arrows indicate autophagic vacuoles. (B) Quantification of the number of autophagic vacuoles per high‐magnification field. (C‐F) LC3 I/II, Beclin‐1 and p62 protein expression at 10 wk after TAC are shown in Western blots and are presented in a bar graph (n = 4). The data are expressed as the mean ± SEM. *P ＜ 0.05, compared to the Sham group; ^#^ *P* ＜ 0.05, compared to the TAC group

We next determined whether the down‐regulatory effect of BZP on autophagy could be observed in vivo subjected to the TAC operation. At 10 weeks after TAC surgery, microscopic images showed that BZP prevented the TAC‐induced increase in the number of autophagic vacuoles (Figure 6A,6). This finding is consistent with the results of Western blot analysis showing that BZP attenuated enhanced LC3 II, Beclin‐1 and impeded the decrease in p62 induced by TAC (Figure 6C,6). These data indicate that BZP attenuated pressure overload‐induced autophagy overactivation in vivo.

### 3.7 BZP suppressed autophagy by suppressing the AMPK‐mTOR signalling pathway

**Figure 7:**
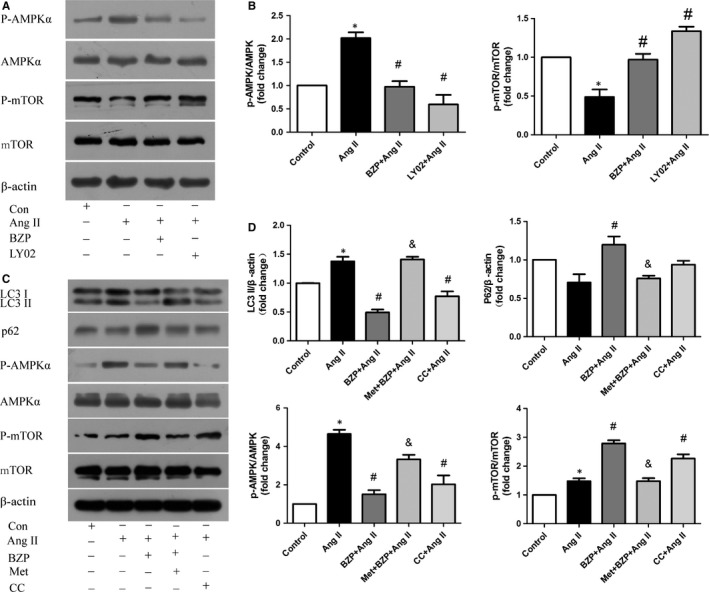
BZP suppressed autophagy by suppressing the AMPK‐mTOR signalling pathway. (A‐B) NRCMs were pre‐treated with or without BZP (250 μM) or LY294002 (5 μM) for 2 h and exposed to Ang II (1 μM) for 48 h. p‐AMPK/AMPK and p‐mTOR/mTOR protein expressions are shown in Western blots and are presented in a bar graph (n = 3). (C‐D) NRCMs were pre‐treated with or without BZP (250 μM) or Compound C (5 μM) for 2 h and exposed to Ang II (1 μM) for 48 h. Metformin (10 mM) was added for 0.5 h before the addition of BZP. The expression of LC3 I/II, p62 and key factors in the AMPK/mTOR pathway are shown in Western blots and are presented in a bar graph (n = 3). The data are expressed as the mean ± SEM. *P ＜ 0.05, compared to the Control group; ^#^ *P* ＜ 0.05, compared to the Ang II group; & *P* ＜ 0.05, compared to the BZP + Ang II group

To elucidate the underlying molecular mechanism and associated signalling pathways of autophagy inhibition by BZP, components of the AMPK‐mTOR signalling pathway were assayed by Western blotting. As shown in Figure 7A,7, significant inhibition of p‐mTOR activity was observed in the Ang II group, indicating that suppression of mTOR contributes to the induction of autophagy by Ang II. In contrast, BZP and LY294002 reversed this process. In addition, BZP and LY294002 reduced the Ang II‐induced phosphorylation of AMPKα, which is an upstream signal of mTORC1. Our results suggested that BZP suppressed Ang II‐induced autophagy, which is mediated by the AMPK‐mTOR signalling pathway.

We further investigated the role of metformin (a specific AMPK activator) and Compound C (a specific AMPK inhibitor) in the BZP‐induced suppression of autophagy. Similar to Compound C, BZP acted as an AMPKα phosphorylation inhibitor and ultimately resulted in autophagy inhibition (Figure 7C,7). Compared to the BZP + Ang II group, metformin reversed the BZP‐induced suppression of autophagy, which was related to its strong activating effect on AMPK phosphorylation (Figure 7C,7). These data suggested that BZP suppressed Ang II‐induced autophagy by suppressing the AMPK‐mTOR signalling pathway.

## 4 DISCUSSION

In this study, we established an in vitro cardiomyocyte hypertrophic model using Ang II treatment as well as a mouse model of myocardial hypertrophy using the TAC operation. Our in vitro data showed that Ang II‐induced cardiomyocyte hypertrophy was accompanied by excessive autophagy (Figure 1). Furthermore, we confirmed that autophagic activity was increased in heart tissue due to the TAC operation (Figure 6). We also demonstrated that BZP inhibited excessive autophagic activity both in vitro and in vivo (Figures 2 and 6), ameliorated cardiomyocyte hypertrophy (Figure 3), alleviated TAC‐induced cardiac hypertrophy and dysfunction (Figures 4 and 5), and suppressed autophagy via suppressing the AMPK‐mTOR signalling pathway (Figure 7). Our results strongly suggest that BZP is a potential drug candidate for treatment of pressure overload‐induced cardiac hypertrophy, and its therapeutic effect might be associated with inhibition of maladaptive cardiomyocyte autophagy.

Autophagy is important in maintaining intracellular homoeostasis because it removes damaged proteins and intracellular organelles,^25^ and it can protect cells in many cases.^26^, ^27^ Nevertheless, excessive or sustained autophagy can lead to accumulation of autophagic products as well as degradation of vital proteins, which can impair cell survival. These conflicting effects have remained a recurrent paradox in the study of autophagy.^28^ In addition, the precise role of autophagy in the haemodynamic stress‐induced model remains controversial. For example, Nakai et al 29 reported that autophagy levels are reduced during the phase of compensated hypertrophy (one week after TAC induction) and are elevated during decompensated heart failure (four weeks after TAC induction). Givvimani et al 7 reported that excessive autophagic activity is induced by pressure overload and that mitophagy inhibition ameliorates pressure overload‐induced heart failure. Cao et al 30 demonstrated that histone deacetylase (HDAC) inhibitors can attenuate cardiac hypertrophy by suppressing autophagy. In our study, electron microscopy of cardiac tissue revealed that the presence of injured mitochondria and autophagic vacuoles was increased subjected to the TAC operation, suggesting impaired energy metabolism and increased oxidative stress.^14^ As a result, autophagy, including mitophagy, was triggered to adapt to the changed circumstances.^31^ However, accompanied by augmenting autophagy, excessive mitochondria degradation led to excessive consumption， thereby aggravating energy metabolism dysfunction.^32^ In contrast, BZP administration attenuated the detrimental autophagic activity and restored the impaired energy metabolism. These data suggest that inhibition of autophagic activity may play a positive role in the therapeutic effect of BZP on pressure overload‐induced cardiac hypertrophy and dysfunction.

To explore the underlying mechanism by which BZP suppressed autophagy, we investigated intracellular signalling pathways. One of the important functions of autophagy is energy recycling.^33^ AMPK is responsible for sensing energy, and it has been proven that activation of AMPK can stimulate autophagy.^34^ Consistent with this finding, we found through Western blotting that AMPK phosphorylation was markedly increased in cardiomyocytes treated with Ang II. By contrast， BZP administration reduced AMPK phosphorylation induced by Ang II. To determine whether BZP attenuated autophagy by suppressing the AMPK pathway, AMPK regulators such as metformin and Compound C were used as positive and negative controls, respectively. We found that metformin reversed BZP‐induced suppression of autophagy, indicating that the autophagy‐inhibiting ability of BZP relied on the inhibition of AMPK phosphorylation.

Our study also has some limitations that should be considered. First, cardiac hypertrophy may be caused by various factors, our study concentrated only on the effects of pressure overload and Ang II administration. Studies on more hypertrophic models are needed to validate our conclusions. Second, the mechanism of drug action is complex, and there must be underlying mechanisms contributing to the therapeutic effect of BZP on cardiac hypertrophy. Finally, as myocardial fibroblasts also play an important role in cardiac remodelling, further investigations are needed to clarify the effect of BZP on cardiac fibroblasts. Such investigations will help deepen the understanding of the potential therapeutic efficacy of this agent against cardiac hypertrophy.

Taken together, for the first time, we demonstrated that BZP could significantly attenuate pressure overload‐induced cardiac hypertrophy and dysfunction, and this effect is associated with the inhibition of maladaptive cardiomyocyte autophagy through the AMPK‐mTOR signalling pathway. Our findings also suggest that BZP may be a good candidate compound for treatment of pressure overload‐induced cardiac remodeling and heart failure.

## CONFLICT OF INTEREST

The authors declare no conflict of interest.

## AUTHOR CONTRIBUTION

JC, JZ, WZ and BW designed the study, researched data, analysed data and drafted the manuscript. DS, JT, JL, YX, XC, CC, DH and EG researched and analysed the data. JZ and WZ provided financial support. All authors read and approved the final manuscript.
